# National Cattle Commission

**Published:** 1881-10

**Authors:** 


THE QUESTION WHETHER WE SHALL HAVE A NATIONAL CATTLE COMMISSION in this country was warmly discussed at a meeting of the Farmers' Club, in this city, last month. Prof. Heath, the president, introduced some resolutions urging the necessity of such a commission. These resolutions were carried by a large majority. There is no doubt that it would be a great benefit to farmers, as well as to owners of live stock, if there were some central body which would keep the country informed regarding contagious diseases among our domestic animals. Exactly how such a body should be constituted remains still to be seen. We have before expressed the opinion that a national commission under the direction of the Agricultural Department would answer the purpose. It would be practically a bureau of that department.

